# Deflection Estimation Based on the Thermal Characteristics of Composite Deck Slabs Containing Macro-Synthetic Fibers

**DOI:** 10.3390/ma14144052

**Published:** 2021-07-20

**Authors:** Dong-Hee Son, Hyo-Jun Ahn, Joo-Hong Chung, Baek-Il Bae, Chang-Sik Choi

**Affiliations:** 1 Department of Architectural Engineering, Hanyang University, Seoul 04763, Korea; son91com@hanyang.ac.kr (D.-H.S.); jun5744@naver.com (H.-J.A.); 2 Department of Smart Architecture Civil Engineering, Daejin University, Pocheon, Gyeonggi-Do 11159, Korea; scarletmoon@daejin.ac.kr; 3 Department of Digital Architectural and Urban Engineering, Hanyang Cyber University, Seoul 04763, Korea; bibae@hycu.ac.kr

**Keywords:** macro-synthetic fiber, steel deck, fire resistance performance, deflection

## Abstract

The purpose of this study was to evaluate the structural performance of composite deck slabs containing macro-synthetic fibers. after a fire by proposing a deflection estimation method for non-fireproof structural decks. Therefore, this study evaluated the fire resistance performance and deflection of deck slabs mixed with macro-synthetic fibers. Afterward, the deflection estimation method considering the thermal characteristics of concrete and deck plates was proposed. A material test was first conducted to evaluate the mechanical properties of concrete mixed with macro-synthetic fibers. This test found that the compressive strength and elasticity modulus of concrete mixed with macro-synthetic fibers was greater than that of general concrete. A flexural tensile test confirmed that residual strength was maintained after the maximum strength was achieved. The fire resistance of the deck slab was adequate even when a fire-resistant coating was not applied. The internal temperature was lowest for the specimen with macro-synthetic fibers. Deflection was evaluated using previously published equations and standards. The deflection evaluation confirmed that the temperature distribution should be applied differently in the estimation method that uses the thermal load of the deck slab.

## 1. Introduction

As buildings have become taller, the number of steel structures has increased. In the past, a steel deck was used only for formwork, in which a reinforcing bar was installed on the steel deck and concrete was poured. However, steel decks have been used recently as structural members, such as a composite structure in which concrete and deck plates are integrated. A composite deck slab is economical because temporary work is unnecessary, and it can even serve as a formwork. Therefore, it is possible to shorten the construction period, increase constructability, and reduce costs [1]. Because the deck plate acts as a tension member, a separate reinforcing bar placement is not required [2,3]. However, the deck slab is vulnerable to high temperatures because the deck plate is exposed to the external environment. To overcome this, fire protection coverings are applied to the exposed surface to form a fire-resistant structure. Modern structural deck plates have a modified shape to increase the composite effect of deck plates and concrete, with the addition of a rib on the bottom steel plate and a Y-shape flange at the end of the rib [2,3]. Because this composite slab increases the composite effect of the deck plate and concrete, fire resistance and structural performance can be ensured without a fire-protective covering on the deck plate [1].

When the composite effect of the deck plate and concrete is increased from the additional rib and Y-shape flange, the deck plate can transfer heat well to the concrete, even when exposed to high temperatures. For this reason, even if the bottom steel plate loses its tensile strength due to high temperatures, it is possible to prevent damage to the deck plate due to the ribs in the web. However, heat is transferred directly into the concrete due to the deck plate rib and Y-shape flange, and the internal temperature of the slab rises compared to the general RC slab. As a result, the water vapor pressure is greater than the internal stress of the concrete, causing spalling [4,5]. Because spalling causes the concrete to fall out, it reduces the strength and usability of the member and causes the structure to collapse. Recent studies have explored the fire resistance of deck plates without fire-protective covering to solve this problem.

**Figure 1:**
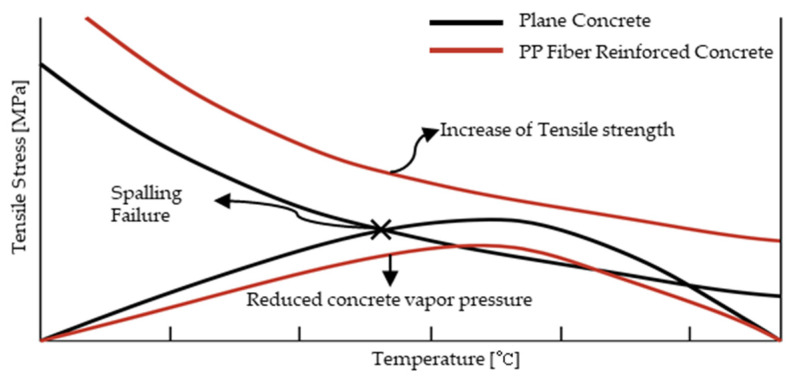
Preventing explosions by applying PP fiber [13].

Researchers have attempted to reduce the internal temperature of concrete through reinforcement with fiber. Fiber-reinforced concrete (FRC) does not require upper temperature shrink reinforcement, so the constructability is expected to increase while the construction process decreases. Macro-synthetic fiber is easy to construct, is more effective at crack control than microfiber, and has high tensile strength [2,3]. Moreover, macro-synthetic fiber is an economical material because it can replace shrinkage and temperature reinforcement and can reduce the reinforcing bar work. According to previous studies, polypropylene (PP) fibers are effective in preventing an explosion of concrete [5,6,7,8]. Macro-synthetic fibers made of polypropylene have a melting point that is approximately 160 °C lower than that of steel or glass fibers. As a result, channels and micro-cracks in which the water can be transported without developing high pore pressures are created. Thus, permeability is observed due to melting of the PP fibers [9]. They form an internal air passage before spalling occurs, which effectively prevents an increase in the internal temperature of the concrete. In a previous study, the permeability of concrete with 1.5 kg/m^3^ PP fibers was shown to be up to four times greater than the permeability of concrete without fibers, and PP fiber contents of 2 kg/m^3^ showed no spalling when flamed [9,10,11]. As shown in Figure 1, spalling can be prevented by decreasing the vapor pressure inside the concrete and increasing the tensile strength [12,13,14].

When evaluating fire resistance performance, the most important thing is whether the structural performance of the member is maintained until the fire resistance certification time determined by domestic and foreign standards [15,16,17,18,19,20]. This was determined in consideration of the evacuation time of users in case of fire.

Accordingly, the deflection of members according to the fire resistance certification time determined by domestic and foreign standards is one of the important factors in evaluating the fire resistance performance, and the method is also presented in various ways through overseas standards and existing studies. Therefore, in this study, the deflection of the member was evaluated in the event of a fire. The thermal change characteristics of the deck plate and concrete were applied according to the temperature; then, the deflection was evaluated by comparing the experimental value and calculated value. The calculated value was based on the material’s mechanical curvature according to the load in the event of a fire and the equation for the fireproof design standard. Through this, the deflection of a deck slab incorporated with macro-synthetic fibers was evaluated and predicted according to the temperature during a fire, which allows for a safety inspection protecting against an actual fire occurrence [15,16,17,18,19,20,21].

## 2. Specimens and Test Methods

### 2.1. Materials

#### 2.1.1. Deck Plate

**Figure 2:**
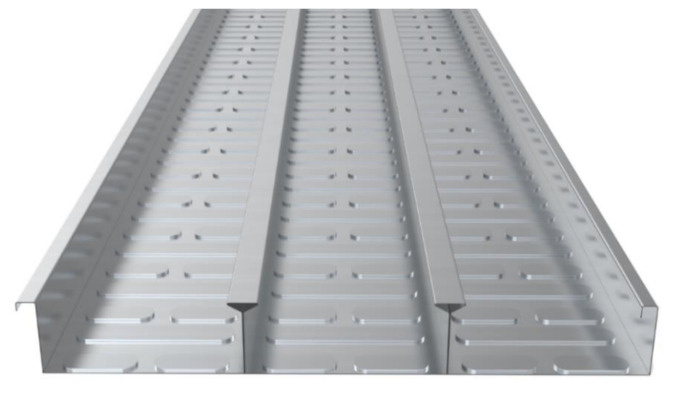
The shape of the deck plate.

The deck plate used in this study was a hot-dip galvanized steel sheet with a product weight of 13.24 kg/m^2^, a cross-sectional area of 13.04 cm^2^, and a thickness of 0.8 mm. The shape and specifications of the deck plate are shown in Figure 2 and Table 1. As shown in Figure 2, by increasing the attach area of the deck plate and concrete through the Y-shaped flange and the embossment of the bottom plate, heat transfer to the concrete was achieved smoothly, even when the deck plate was exposed to high temperatures. To confirm the effect of the deck plate, a general reinforced concrete slab specimen was also manufactured with the same amount of reinforcement as the deck plate. Through this, the effect of the thermal properties of concrete on the deck was compared and analyzed with the general reinforced concrete slab specimen.

#### 2.1.2. Characteristics of the Macro-Fiber

**Figure 3:**
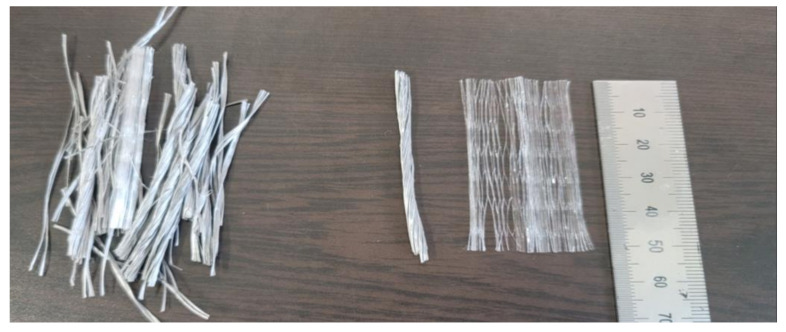
Macro-synthetic fiber [2].

The macro-synthetic fibers were polypropylene-based synthetic fibers that were 54 mm long, 0.34 m in diameter, and 159 in fiber shape ratio, which was used to confirm control of the explosion. The mechanical properties of macro-synthetic fibers were taken from the manufacturer’s data [22]. The tensile strength was 54.9 MPa and the modulus of elasticity was 4.7 GPa. The shape and details of the fiber are shown in Table 2 and Figure 3. The macro-synthetic fiber was used by mixing twisted and fibrillated fiber types in a ratio of approximately 9:1 [2]. Because macro-synthetic fibers have extremely hydrophobic properties because of their chemical structure, their agglomeration is less than that of other fibers when concrete is mixed [22,23]. In previous research, the mixed fibers presented even rougher and more irregular surfaces due to the abrasion caused by the mixing with the aggregates. Because of this it can be observed that the macro-synthetic-fiber-specific surface area increased in the process when compared with the original fiber. Therefore, the cement hydration products could penetrate in between the filaments, promoting a higher adherence between the macro-synthetic fiber and matrix [24].

### 2.2. Material Test

The test variables for the deck slab test specimen were the presence or absence of a deck plate and the presence of macro-synthetic fibers. The concrete mix proportion table used in the deck slabs is shown in Table 3. The concrete compressive strength test was performed according to KS F 2405 [25]. On the 28th day, the compressive strength of the specimen was measured to be 34.2 MPa on average for the specimen without fibers and 33.1 MPa for the specimen containing macro-synthetic fibers. In addition, the average compressive strength of the specimen on the 90th day, which was the fire resistance test day, was 37.6 MPa for the specimen without fiber and 26.6 MPa for the specimen with macro-synthetic fiber; the compressive strength was lower when the fibers were mixed. For the modulus of elasticity, the slope for the compressive stress of concrete corresponded to 0.45 $f_{c}^{\prime }$ [21]. The average elastic modulus was confirmed to increase when fiber was incorporated. Table 4 shows the results of the compressive strength test.

A flexural tensile test was performed with a three-point loading force by making a notch in the center of the specimen according to KS F 2408 [26] and fib Model Code 2010 [27], as shown in Table 5. On the 28th day, the flexural strength of the specimen was measured to be 3.51 MPa on average for the specimen without fibers and 3.7 MPa for the specimen containing macro-synthetic fibers. In order to confirm the tensile resistance effect due to fiber mixing, the residual stress according to the crack mouth opening displacement (CMOD) during three-point loading was measured. In fib Model Code 2010 [27], tensile stress is measured when the CMOD is 0.5 mm, 1.5 mm, and 3.5 mm, respectively. As a result, although the difference in the maximum flexural strength was small, the residual strength was maintained because a tension-softening effect appeared after the maximum tensile strength was achieved. Thus, the macro-synthetic fibers appear to be effective at increasing the flexural strength. [2]. The deck plate used in the tests was SGC 365Y steel grade, as suggested in KS D 3506 [28]. The yield strength was 365 MPa and the modulus of elasticity was 210,000 MPa [2].

### 2.3. Test Plan and Specimen Design

**Figure 4:**
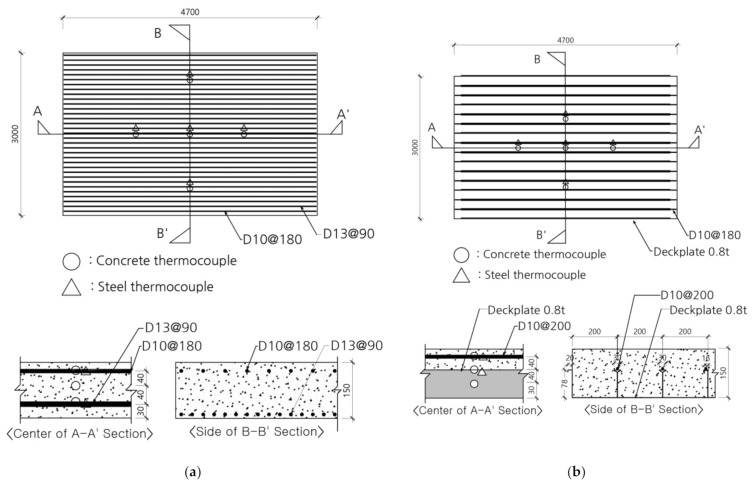
Details of the specimens and location of the thermocouple (unit: mm). (**a**) RC-0. (**b**) Deck-0, 2.4.

The deck slab specimen on which the fire resistance performance test was performed was manufactured in a rectangular shape with a size of 4700 mm × 3000 mm, including a 250-mm support at each end, as shown in Figure 4 and Table 6. Thermocouples were installed at heights of 30 mm, 80 mm, and 120 mm on the concrete, upper and lower reinforcing bars, and deck plates, as shown in Figure 4, to determine the internal temperature of the test specimen [29,30,31].

The test specimen was cured for 3 months after pouring so that the moisture content of the concrete was suitable for the conditions of use of the slab. The fire resistance test was carried out in air-dry conditions.

### 2.4. Test Method and Setup

The fire-resistance performance test was conducted according to the KS F 2257-1 [19] fire-resistance performance test method and ISO-834 [17]. All of the specimens were heated for 120 min. The lower surface of the slab was heated according to the ISO-834 standard time-temperature curve in Equation (1):

(1) $$T_{\left(t\right)}=345\;\cdot \;\log\left(8t+1\right)T_{0}$$

Here,

*t*: Fire occurrence time (min)
$T_{\left(t\right)}$: Average temperature in the heating furnace (°C)
$T_{0}$: Air temperature (20 °C)

The fire-resistance performance evaluation of the test specimen was evaluated under the three conditions of load-bearing capacity, heat-shielding properties, and flame protection specified in the fire test method for KS F 2257-1 [19]. When the load-bearing capacity evaluation exceeds the limit deformation and allowable deformation rate according to the thickness of the slab, the member is determined to have collapsed and the maximum deflection criterion is not exceeded. Because sudden deformation may occur until the specimen reaches a steady state, the performance criterion for the deformation rate is not applied until the deformation amount exceeds L/30. KS F 2257-1 is judged to be suitable when both criteria are satisfied through Equations (2) and (3) for limit deformation:

(2) $$\begin{array}{cc} \mathrm{Limit}\;\mathrm{deformation} & D=\frac{L^{2}}{400d}\left(\mathrm{mm}\right) \end{array}$$

(3) $$\begin{array}{cc} \mathrm{Allowable}\;\mathrm{deformation}\;\mathrm{rate} & \frac{dD}{dt}=\frac{L^{2}}{9000d}\;\left(\mathrm{mm}/\min\right) \end{array}$$

Here,

L: Span (mm)
d: The distance from the position designed to receive the maximum compressive force of the structural section to the position designed to receive the maximum tensile force (mm)

**Figure 5:**
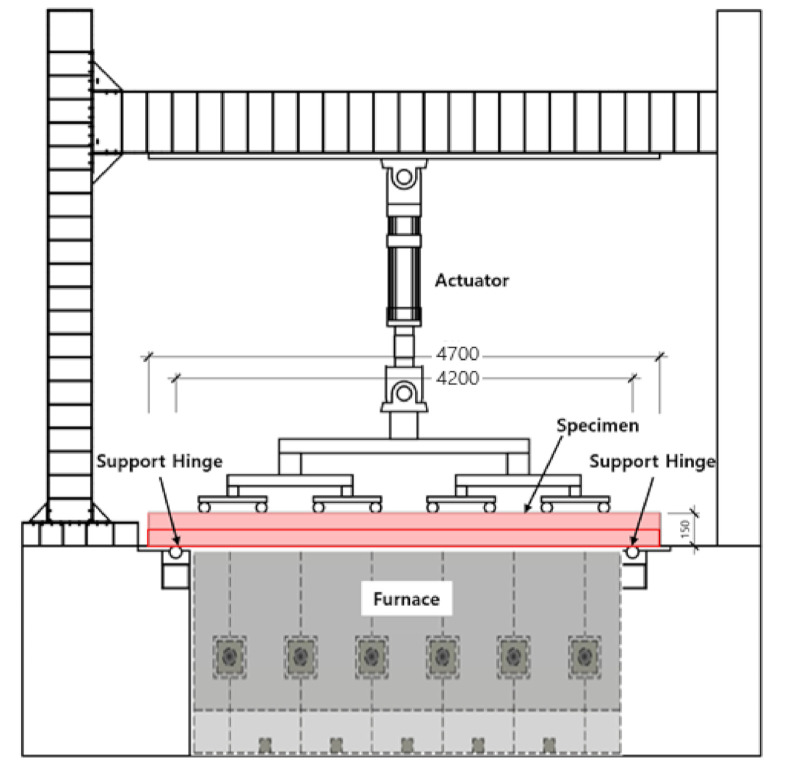

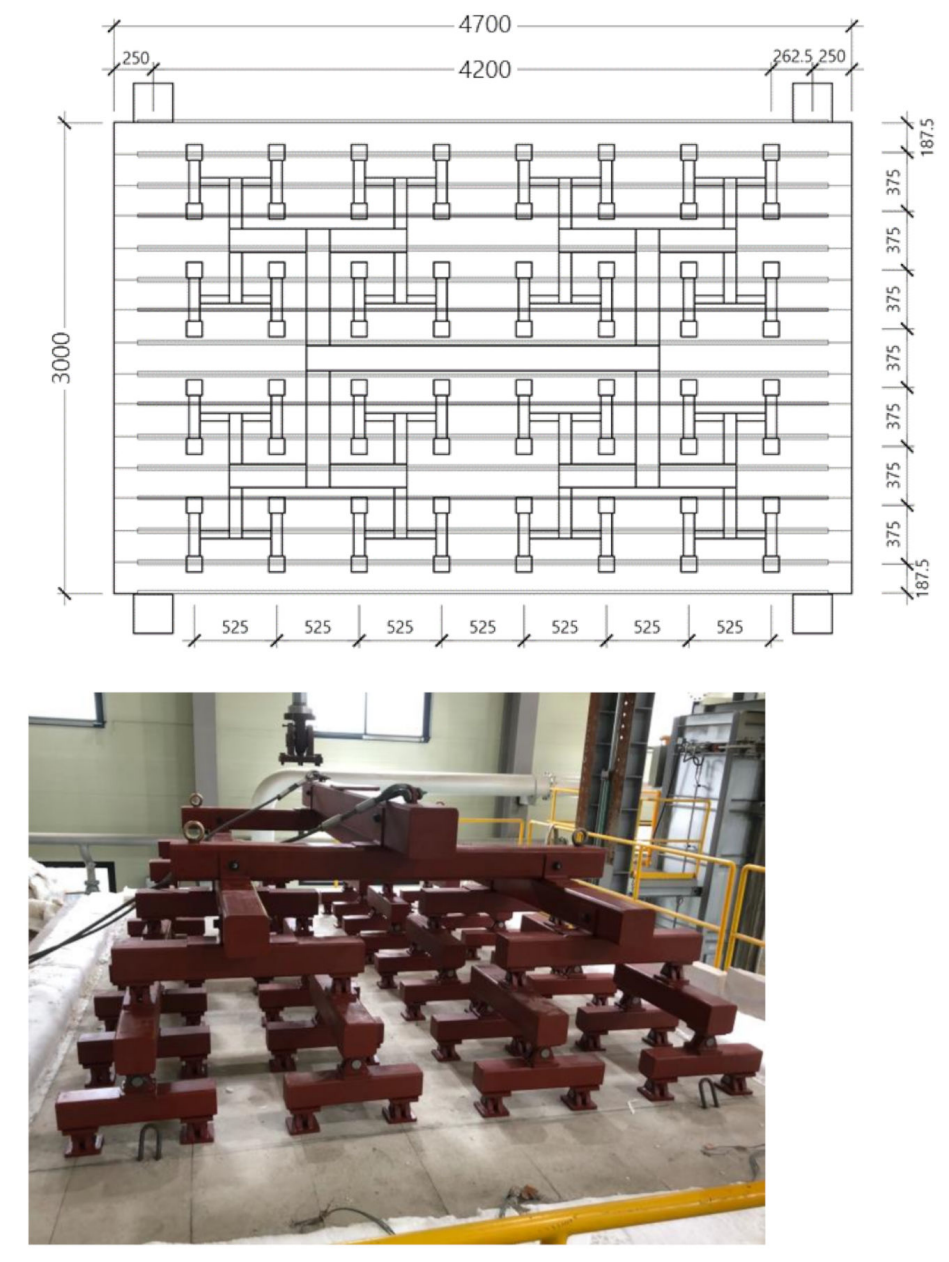
Fire-resistance test specimen loading and installation (unit: mm).

In addition, if the temperature of the non-heating surface of the test body increased by more than 140 K from the initial temperature during the fire test, it was judged as unsuitable in terms of heat-shielding performance. To evaluate the fire resistance, the test was performed by loading the upper part of the test sample with an equal distribution load according to the fire test standard, as shown in Figure 5. In the case of the total load, 90 kN (which is approximately 34.7% of the nominal strength of 205 kN·m) was loaded; the uniform load was 9 kN/m^2^, including the weight of the loading fixture.

## 3. Test Results

### 3.1. Deflection Change According to Temperature

**Figure 6:**
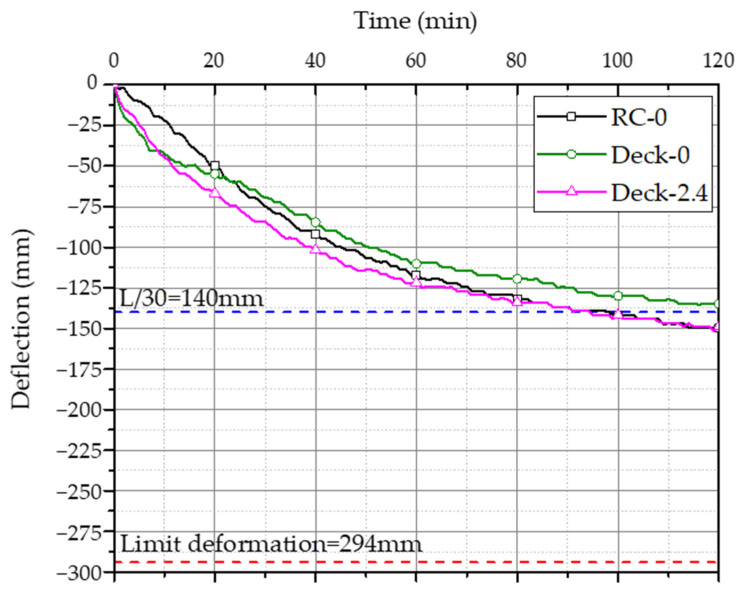
Deck slab deflection–time curve.

The fire-resistance performance test results are shown in Table 7. Figure 6 shows the amount of deformation of each specimen over time. The standard of the limiting amount of deformation according to Equation (2) is indicated by a red dotted line. The Deck-0 test specimen did not exceed the limit strain of L/30 = 140 mm, but the RC-0 and Deck-2.4 specimens exceeded the limit strain of L/30 = 140 mm. Therefore, the fire resistance was evaluated in terms of L/9000d, which is the allowable strain standard for structural safety. When evaluating the load-bearing capacity, the RC-0 specimen did not exceed 294 mm based on the limit deformation amount or 13 mm/min based on the allowable deformation rate, with a deformation amount of 149.8 mm and a deformation rate of 5.7 mm/min during the fire-resistance time target of 120 min. In addition, in the case of the Deck-0 test specimen, the strain was 134.8 mm and the strain was 8.0 mm/min, which did not exceed the limit and allowable strain standards. In the case of the Deck-2.4 specimen, the strain was 151.5 mm and the strain was 6.6 mm/min, which satisfies both the limiting strain and the allowable strain.

In terms of heat-shielding properties, the non-heating surface temperature averaged 79.4 °C and the maximum was 102.1 °C for the RC-0 test specimen. None of the three test specimens exceeded the standard for backside rise temperature. In the fire-resistance performance test, none of the three specimens exceeded the standard values for the deformation, strain, and non-heating surface elevation temperature; they satisfied the fire resistance recognition time of 120 min for floor members, as specified in the fire resistance structure standard.

When comparing RC-0 and Deck-0, the amount of deformation of Deck-0 decreased by 15 mm compared to RC-0. In general, when the internal temperature of concrete rises, the pressure of the internal aggregate itself increases, causing the concrete to expand and consequently deteriorating the performance of the concrete [13]. However, in these test results, the flexural strengths of RC-0 and Deck-0 were the same, although the flexural strength of Deck-0 was determined to be higher than that of RC-0 because of the ribs and Y-shape flanges in the web. Deck-2.4 showed the largest amount of deformation compared with RC-0 and Deck-0; the internal temperature change was determined to explore this in more detail.

It was confirmed that bending cracks appeared in the center of all specimens. It is judged that crack occurrence and deflection changed despite no change in load as the flexural strength and stiffness decreased as the temperature of concrete, reinforcing bars, and deck plates increased.

### 3.2. Thermal Properties of the Deck Slabs

**Figure 7:**
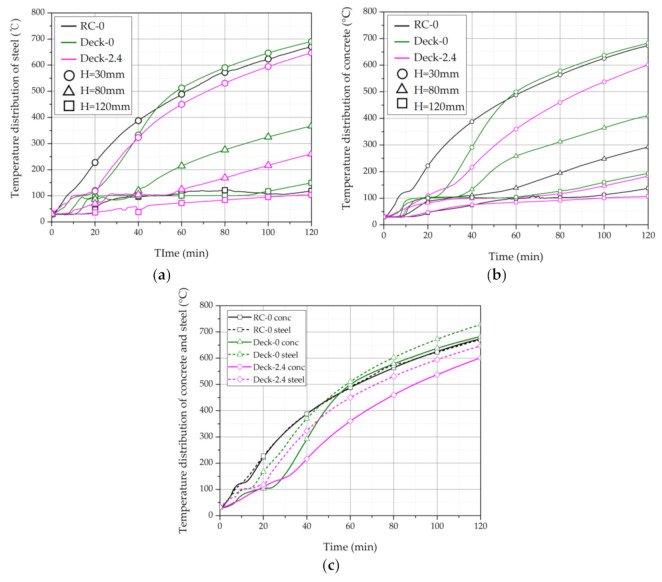
Internal temperature distribution. (**a**) Rebar and deck plates. (**b**) Concrete. (**c**) Concrete and steel temperatures at the 30-mm position.

The internal temperature distribution for each specimen is shown in Figure 7 and Table 8. As shown in Figure 7a,b, when examining the temperature distribution of the steel (rebar) and concrete of the 30-mm-high Deck-0 and Deck-2.4 specimens, both specimens showed a delayed period of temperature rise between approximately 10 and 20 min. This delay in temperature rise in a specific section is a phenomenon that is difficult to see in general reinforced concrete slabs. In the deck slab specimen, the delay period for the initial temperature was determined to occur when the specimen was heated because the evaporation of water during the curing period was less than RC-0 due to the deck plate at the bottom of the slab.

Between 20 and 60 min after the start of the fire resistance test, the temperature of the 30-mm deck slab specimen rapidly increased, then gradually changed after 60 min to show a similar slope to the RC-0 specimen. In the case of general concrete, the thermal conductivity of the concrete decreased due to a decrease in the mass of the concrete and the creation of voids as the temperature increased and the moisture inside escaped. In the case of the deck plate, after the initial moisture evaporation on the concrete’s bottom surface, heat transfer occurred actively, showing a rapid temperature increase between 20 and 60 min. After that, when the concrete reached a certain temperature, a temperature pattern similar to that of RC-0 appeared.

To determine the heat transfer effect by the deck plate, the temperatures of the rebar at the 30-mm position and the concrete were compared, as shown in Figure 7c. In the case of RC-0, the temperature difference did not occur as the concrete directly transferred heat to the reinforcing bar. In the case of Deck-0, as mentioned above, the increase in the concrete temperature was delayed due to the evaporation of moisture, resulting in a temperature difference between the deck and the concrete that received heat directly.

When checking the temperature change at the top, the temperature at 120-mm height inside the concrete was 134.3 °C on average in the Deck-0 test specimen compared with 114.6 °C for the RC-0 specimen and 102.6 °C for the Deck-2.4 specimen at the same location. It was measured at the highest temperature. This temperature difference is thought to be caused by smooth heat transfer from the deck plate to the concrete as heat rises through the ribs due to the ribs in the abdomen of the deck plate [32,33].

### 3.3. Thermal Properties of Deck Slabs with Macro-Synthetic Fibers

As shown in Figure 7b, most of the temperatures of Deck-2.4 were lower than those of Deck-0 due to the mixing of macro-synthetic fibers. In particular, the average temperature difference of the upper part was 12 °C lower than that of the RC-0 test specimen, with an average temperature of 114.6 °C for the RC-0 test specimen but 102.6 °C at the 120-mm position inside the concrete.

As the fibers were mixed, the lowest temperature was found in the bottom surface of the specimen. According to a previous study [5], polypropylene fibers inside concrete melt when exposed to high temperatures during a fire test, forming a water vapor passage. Air circulation through this passage evenly increases the internal temperature of the test body [5]. Because the main material of the macro-synthetic fiber in this study was also polypropylene, test results similar to those of previous studies were obtained [34,35,36].

**Figure 8:**
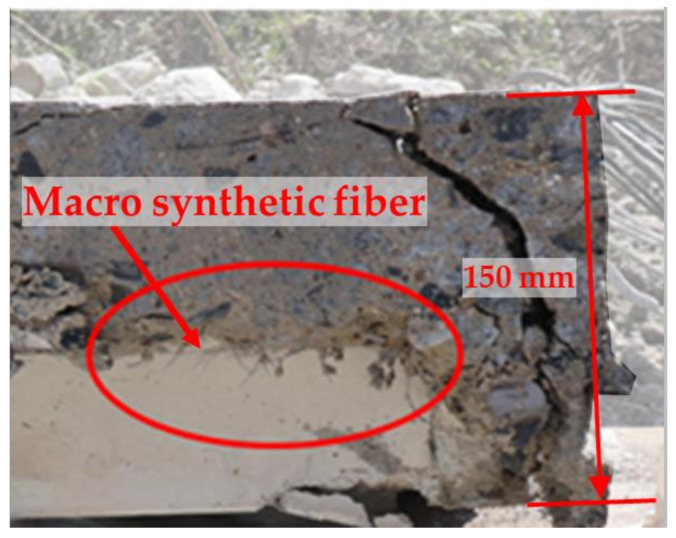
Section of Deck-2.4.

When checking the cross-section of Deck-2.4 after the fire test was completed, as shown in Figure 8, all fibers with a height of 80 mm or more in the deck plate were intact. As shown in Figure 7 and Table 8, the temperature at the 120-mm position of Deck-2.4 was an average of 102.6 °C, which is less than the melting point of the polypropylene fibers (160 °C).

## 4. Deflection Estimation of Composite Deck Plate Slab Exposed to High Temperatures

In case of fire, slab deflection evaluation requires analytical evaluation applying material properties. In this study, the deflection evaluation was performed through the deflection estimation formula presented in the previous study based on the temperature distribution derived from the experiment. When evaluating deflection by analysis, as shown in Equation (4), the total deflection of the member is expressed as the sum of deflection due to external load and deflection caused by temperature, which is calculated under the assumption of two conditions [31]:

(4) $$\delta_{total}=\delta_{load}+\delta_{thermal}$$

- The sum of compressive stresses occurring in the member is always equal to the sum of the tensile stresses.
- The moment generated by compression and tension force is the same as the applied moment.

The neutral axis can be obtained based on the above two conditions. After calculating the strain through this method, the stress is calculated using the stress–strain equation. The nominal moment can be obtained from this stress, and the actual curvature can be considered when it is equal to the moment caused by the external load.

Because the deflection of the member due to temperature increase is not due to the temperature load, the deflection equation obtained by integrating twice the moment cannot be used directly. Therefore, Equations (5) and (6) were obtained based on the fact that the slope of the linear strain and the curvature of the members are the same, and that the value obtained by dividing the moment by the flexural stiffness is the same as the curvature:

(5) $$\delta =\iint \phi d_{z}=\frac{1}{2}\phi \cdot z^{2}+C_{1}\cdot z+C_{2}=\frac{1}{2}\phi \cdot z^{2}-\frac{1}{2}L\phi \cdot z$$

(6) $$\delta_{\max\left(z=\frac{L}{2}\right)}=-\frac{1}{2}\phi \cdot {\left(\frac{L}{2}\right)}^{2}$$

Deflection due to thermal load was calculated using two assumptions—one caused by the moment at the end and one caused by the axial force at the end. To quantitatively evaluate deflection during a fire for a deck slab incorporated with macro-synthetic fibers, the creep strain based on the concrete temperature change according to the Anderberg model [20] and the thermal strain of the reinforcing bar as proposed by Eurocode 2 were applied [15]. The strain of concrete is equal to Equation (7) and the thermal strain of reinforcement is equal to Equation (8):

(7) $$\begin{array}{l} \mathrm{For}\;20\;{}^{\circ}C\leq T\leq 700\;{}^{\circ}C,\\ \;\varepsilon_{th}=-\left(-1.8\times 10^{-4}+9\times 10^{-6}T+2.3\times 10^{-11}T^{3}\right)\\ \mathrm{For}\;700\;{}^{\circ}C\leq T\leq 1200\;{}^{\circ}C,\\ \;\varepsilon_{th}=-14\times 10^{-3} \end{array}$$

(8) $$\varepsilon_{th}\left(T\right)=-2.416\times 10^{-4}+1.2\times 10^{-5}T+0.4\times 10^{-8}T^{2}$$

When assuming that the deflection caused by the thermal load occurs due to the moment at the end, it can be calculated according to the general curvature derivation process. A case in which deflection occurs in a simply supported member is shown in Equations (9) and (10):

(9) $$\upsilon_{\phi ,th}=\frac{\phi_{th}}{2}x^{2}+\frac{\phi_{th}}{2}lx,\;\;\;\phi_{th}=-\frac{\epsilon_{total}\left(T\right)}{h}$$

(10) $$\epsilon_{total}\left(T\right)=\epsilon_{th}+\epsilon_{creep}$$

Here, $\phi_{th}$ is the curvature due to thermal load, x is the distance from the end of the member to the center, *l* is the length of the member, $\epsilon_{total}\left(T\right)$ is the strain due to thermal load, and h is the depth of the measured temperature change.

Second, when assuming that the deflection caused by the thermal load is caused by the axial force at the end, it can be calculated according to Equation (11). This method measures the deflection of the member due to thermal load according to the deformation of the main reinforcing bar of the member and shows the deflection equation according to the axial force [18]:

(11) $$\upsilon_{\phi ,th}=\frac{2l}{\pi }\sqrt{\epsilon_{total}\left(T\right)+\frac{\epsilon_{total}\left(T^{2}\right)}{2}}$$

**Figure 9:**
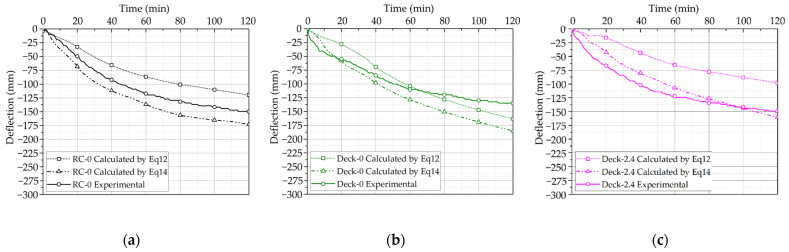
Deflection of specimens. (**a**) Deflection of RC-0. (**b**) Deflection of Deck-0. (**c**) Deflection of Deck-2.4.

Figure 9 compares the proposed deflection calculation methods with the experimental results. The measured internal temperature of the specimen was applied differently in each of the proposed deflection calculation methods. First, when assuming that the thermal load was generated by the moment at the end of the specimen, the temperature changes of the lower reinforcing bar and upper concrete had the greatest influence on the deflection calculation. Therefore, when calculating the curvature due to temperature, the average value of the temperature distribution measured by the thermocouple located on the lower surface of the slab was used for the strain due to the reinforcing bar, and the average of the temperature measured by the upper concrete thermocouple was used for concrete. When assuming that the thermal load was generated by the end axial force, the average of 15 thermocouples measured at three heights and five positions for both concrete and reinforcement was applied.

Considering the results of the deflection estimations for all specimens, the equation based on axial force more conservatively evaluated fire resistance performance than did the equation based on bending. The deflection estimation method described above is a method applied to a general RC slab. The actual deflection falls between the estimated deflection due to axial force and the estimated deflection due to bending.

In the case of Deck-0, the internal temperature of the specimen was the highest among all specimens. However, the structural performance was expressed according to the composite of the deck plate and concrete to prevent deformation. In the case of Deck-2.4, the internal temperature of the specimen was the lowest among all specimens. The estimated sag was also the smallest because the rear surface temperature was reduced by up to approximately 30 °C and 90 °C by the macro-synthetic fiber compared with the other specimens. However, when compared with the actual experimental results, the estimated value of deflection due to bending was approximately 50 mm greater; when compared with the estimated value of deflection due to axial force, it was approximately 12 mm smaller.

If the estimation of the temperature distribution considering the thermal characteristics of the deck slab and macro-synthetic fiber is derived in the future, the deflection of the slab in case of fire can be derived analytically without relying on experiments by applying the deflection evaluation method performed in this study.

## 5. Conclusions

This study examined the thermal properties of non-fireproof deck slabs and macro-synthetic fibers. A total of three test specimens were produced: general reinforced concrete slabs, deck slabs, and deck slabs containing 2.4 kg/m^3^ of macro-synthetic fibers. A fire resistance test was conducted according to KS F 2257 [19]. Through this study, a method for estimating the deflection of the deck plate incorporated with macro-synthetic fibers was derived without conducting a fire resistance test, and the conclusions are as follows:

(1) Based on the results of a fire test on the deck slab, RC-0, Deck-0, and Deck-2.4 all satisfied the fire resistance certification time of 120 min for the floor member.
(2) Based on an analysis of the temperature distribution of the deck slab, the temperature increase was delayed in a section after a certain time, which is believed to have affected the overall temperature increase.
(3) The internal temperature of Deck-0 was the highest compared with other specimens, but the deformation was the lowest. This is believed to have reduced the deformation of the deck plate because the composite effect increased due to the ribs arranged in the web and the Y-shape flange at the end of the ribs.
(4) Based on an analysis of the thermal properties of the macro-synthetic fibers, Deck-2.4 (containing macro-synthetic fibers) had the lowest backside temperature compared with the other test specimens with a deformation difference of 16.7 mm compared with Deck-0. Therefore, the incorporation of macro-synthetic fibers is effective in preventing an increase in the temperature inside the slab, although it did not cause any reduction in the amount of deformation.
(5) When estimating deflection due to thermal load for the deck slab, the temperature distribution should be applied differently for each method of estimating deflection. In addition, the method for estimating deflection due to axial force was found to safely evaluate the fire resistance performance of a deck slab.
(6) If the estimation of the temperature distribution considering the thermal characteristics of the deck slab and macro-synthetic fiber is derived in the future, the deflection of the slab in case of fire can be derived analytically without relying on experiments by applying the deflection evaluation method performed in this study. In addition, it is necessary to analyze the effect of the length and angle of the rib and the Y-shape on the fire resistance performance of the deck plate later through finite element analysis.

## Figures and Tables

**Table 1:** Deck plate sectional performance.

Thickness (mm)	Weight (kg/m^2^)	Section Area (mm^2^)	Centroid (mm)
0.8	14.47	1075.2	25.29

**Table 2:** Fiber specifications [2,3].

Material	Tensile Strength (MPa)	Modulus of Elasticity (GPa)	Length (mm)	Diameter (µm)	Aspect Ratio
Polypropylene	54.9	4.7	54	0.34	159

**Table 3:** Mix proportions of the concrete.

W/C (%)	S/a (%)	Unit Weight (kg/m^3^)					
		C	W	S	G	AD	MF
54.9	45.5	365	200.7	854	1022.7	3.29	0
							2.4

W/C: water-cement ratio, S/a: fine aggregate ratio, C: cement, W: water, S: fine aggregate, G: coarse aggregate, AD: admixture, MF: macro-synthetic fiber.

**Table 4:** Concrete compressive test results.

Day	No.	$V_{f}$ (kg/m^3^)	$f_{c}^{\prime }\;(\mathrm{MPa})$	$\varepsilon_{0}\;(\mathrm{mm}/\mathrm{mm})$	$E_{c}\;(\mathrm{MPa})$
28	1	0	34.70	0.00163	38,912
	2		34.98	0.00167	35,457
	3		35.74	0.00143	41,128
90	1		37.6	0.00158	32,696
	2		38.49	0.00169	37,551
	3		39.41	0.0018	32,245
28	1	2.4	39.35	0.00164	42,408
	2		40.94	0.00140	46,054
	3		39.89	0.00194	32,934
90	1		36.19	0.00173	35,558
	2		33.89	0.00128	38,126
	3		26.77	0.00164	33,463

$V_{f}$: macro fiber dosage, $f_{c}^{\prime }$: compressive strength of concrete, $\varepsilon_{0}$: strain at peak stress, $E_{c}$: modulus of elasticity.

**Table 5:** Concrete tensile test results.

Day	No.	$V_{f}$ (kg/m^3^)	$f_{r}\;(\mathrm{MPa})$	$f_{R,1}\;(\mathrm{MPa})$	$f_{R,2}\;(\mathrm{MPa})$	$f_{R,3}\;(\mathrm{MPa})$
28	1	0	3.708	-	-	-
	2		3.308	-	-	-
	3		-	-	-	-
	1	2.4	3.456	1.252	1.204	1.104
	2		3.708	0.904	0.904	0.706
	3		3.906	0.754	0.704	0.704

$V_{f}$: macro fiber dosage, $f_{r}$: flexural tensile stress, $f_{R,1,2,3}$: residual flexural tensile strength corresponding to CMOD = 0.5 mm, 1.5 mm, and 2.5 mm.

**Table 6:** Test specimen details.

Specimen	Thickness (mm)	Length (m)	Width (m)	Macro-Synthetic Fibers (kg/m^3^)	Concrete Cover Thickness (mm)		Top Rebar	Bottom Rebar	Uniform Load (kN/m^2^)
					Top	Bottom			
RC-0	150	4.7	3.0	0	20	20	D10@200	D13@90	7.2
Deck-0				0		-		-	
Deck-2.4				2.4		-		-	

**Table 7:** Fire resistance performance of the deck slab.

Specimen	Displacement (mm)		Rate of Displacement (mm/min)		Temperature Increase at Unheated Surface (°C)		Fire Resistance Performance (O/X)
	Limitation	Measured	Limitation	Measured	Average	Maximum	
RC-0	294	149.8	13	5.7	79.4	102.1	O
Deck-0	294	134.8	13	8.0	99.3	158.4	O
Deck-2.4	294	151.5	13	6.6	65.0	69.8	O

**Table 8:** Internal temperature of slab by specimen.

Specimen	Measurement	Temperature (°C) at 120 min					
		Rebar and Deck Plate			Concrete		
		30 mm	80 mm	120 mm	30 mm	80 mm	120 mm
RC-0	Average	582.6	-	115.3	487.8	225.3	114.6
	Maximum	670.4	-	149.0	673.2	292.5	137.6
Deck-0	Average	642.0	292.6	116.8	480.5	255.4	134.3
	Maximum	728.3	367.3	149.3	682.8	410.2	193.4
Deck-2.4	Average	609.8	292.0	101.1	458.8	189.5	102.6
	Maximum	646.8	379.2	103.6	601.7	237.7	107.4

## Data Availability

For data is contained within the article.
